# Toward a causal link between attachment styles and mental health during the COVID‐19 pandemic

**DOI:** 10.1111/bjc.12428

**Published:** 2023-06-09

**Authors:** Laura M. Vowels, Matthew J. Vowels, Katherine B. Carnelley, Abigail Millings, Jilly Gibson‐Miller

**Affiliations:** ^1^ Department of Social and Political Sciences, FAmily and DevelOpment Research Centre (FADO), Institute of Psychology University of Lausanne Lausanne Switzerland; ^2^ Department of Social and Political Sciences, Cognitive and Affective Regulation Laboratory (CARLA), Institute of Psychology University of Lausanne Lausanne Switzerland; ^3^ School of Psychology University of Southampton Southampton UK; ^4^ Department of Psychology, Sociology and Politics, Centre for Behavioural Science and Applied Psychology Sheffield Hallam University Sheffield UK; ^5^ School of Education University of Sheffield Sheffield UK

**Keywords:** attachment style, COVID‐19, loneliness, mental health, social distancing behaviours

## Abstract

**Background:**

Recent research has shown that insecure attachment, especially attachment anxiety, is associated with poor mental health outcomes, especially during the COVID‐19 pandemic. Other research suggests that insecure attachment may be linked to nonadherence to social distancing behaviours during the pandemic.

**Aims:**

The present study aims to examine the causal links between attachment styles (secure, anxious, avoidant), mental health outcomes (depression, anxiety, loneliness) and adherence to social distancing behaviours during the first several months of the UK lockdown (between April and August 2020).

**Materials & Methods:**

We used a nationally representative UK sample (cross‐sectional *n* = 1325; longitudinal *n* = 950). The data were analysed using state‐of‐the‐art causal discovery and targeted learning algorithms to identify causal processes.

**Results:**

The results showed that insecure attachment styles were causally linked to poorer mental health outcomes, mediated by loneliness. Only attachment avoidance was causally linked to nonadherence to social distancing guidelines.

**Discussion:**

Future interventions to improve mental health outcomes should focus on mitigating feelings of loneliness. Limitations include no access to pre‐pandemic data and the use of categorical attachment measure.

**Conclusion:**

Insecure attachment is a risk factor for poorer mental health outcomes.

## BACKGROUND

The COVID‐19 pandemic has brought many challenges including navigating how best to protect our health and well‐being, while living our lives to the fullest. For some, the circumstances surrounding COVID‐19 have been more detrimental to their mental health than for others (Shevlin et al., 2021). In this paper, we test novel hypotheses with important implications for well‐being using data from early in the pandemic collected by the COVID‐19 Psychological Research Consortium Study (C19PRCS), a longitudinal survey tracking changes in behaviour and mental health over the pandemic in a large representative sample of the UK adult population. We aimed to develop a theoretical causal model to better understand how individual differences in attachment styles influence adherence to social distancing behaviours, as well as mental health outcomes (loneliness, depression, and anxiety) in the context of the pandemic. Importantly, we used a cutting‐edge causal discovery algorithm known as Structural Agnostic Modelling (SAM; Kalainathan et al., 2020) to estimate the causal structure of the model. We then estimated and tested specific causal effects within the model using targeted learning (van der Laan et al., 2007). Using these advanced methods allowed us to examine the possible causal relationships between attachment styles, social distancing behaviours, and mental health outcomes during the pandemic in a representative sample of UK adults.

### Mental health during the COVID‐19 pandemic

The pandemic has led to an increase in mental health difficulties in many nations (Burkova et al., 2021; Pierce et al., 2020; Randall et al., 2022). For example, a systematic review of 43 cross‐sectional studies (Vindegaard & Benros, 2020) showed higher rates of depression, anxiety, and posttraumatic stress‐disorder (PTSD) compared to before the pandemic. Several longitudinal studies support this pattern, for example, Pierce et al. (2020) compared pre‐pandemic levels to 1 month into lockdown in the UK and showed roughly a 10% increase in depression and anxiety after the pandemic began. Other longitudinal studies that examined outcomes over the course of the pandemic show mixed results. Huckins et al. (2020) found an increase in anxiety and depression over 12 weeks during the pandemic in a student sample, whereas Wang et al. (2020) found that the moderate to severe levels of stress, depression, and anxiety assessed at the start of the pandemic and 4 weeks later in China initially did not change over this time.

### Attachment styles and mental health during the COVID‐19 pandemic

Although many people have found the COVID‐19 pandemic stressful, not everyone has experienced worse mental health (e.g., Shevlin et al., 2021). Research has examined several factors that predict who is more likely to experience elevated depression, anxiety, and poor well‐being due to the pandemic. One such variable is adult attachment style, which describes the ways people think, feel, and behave in their close relationships that are developed based on experiences with caregivers and are mentally represented as internal working models. The primary purpose of forming these ties or ‘attachments’ is to maintain proximity to our caregivers and thus ensure survival (Bowlby, 1969). The quality of these attachment relationships throughout life becomes internalized over time as ‘attachment styles’ (Bowlby, 1969; Brennan et al., 1998). When an individual experiences sensitive, responsive care from their attachment figures (parents and partners), they develop attachment security. Attachment security is associated with happiness, life satisfaction, and more positive physical and mental health and well‐being outcomes (Mikulincer & Shaver, 2016a, 2016b). Conversely, a history of experiences in which attachment figures are rejecting, or inconsistent, leads to attachment insecurity, conceptualized as avoidance (of intimacy) or anxiety (about abandonment; Brennan et al., 1998). These individual differences should theoretically link to the ability to adapt behaviourally and emotionally to the demands enforced by the COVID‐19 pandemic.

Indeed, research demonstrates that adult attachment styles have influenced well‐being during the COVID‐19 pandemic. Moccia et al. (2020) found that attachment anxiety differentiated between those who reported moderate–severe psychological distress versus no distress during the pandemic in Italy, suggesting that attachment anxiety is a risk factor for mental health problems. Similarly, Mazza et al. (2021) found in an Italian sample of healthcare workers that attachment anxiety was positively associated with high stress, depression, and anxiety. Carbajal et al. (2021) also focused on healthcare workers and found that both attachment anxiety and avoidance were negatively associated with resilience and positively associated with depression and PTSD, whereas attachment anxiety was positively associated with generalized anxiety and suicidality. Building on this cross‐sectional work, Vowels, Carnelley, and Stanton (2022) examined the effects of adult attachment on depression and anxiety in two longitudinal studies that assessed depression and anxiety weekly over 5 weeks near the start of lockdown in the UK (Study 1) and compared the levels of depression and anxiety from pre‐pandemic to during pandemic (Study 2). The authors found that those high in attachment anxiety experienced higher depression and anxiety than those low in attachment anxiety. Furthermore, those higher in attachment anxiety maintained their elevated levels of depression and anxiety across the 5 weeks, but those lower in attachment anxiety reported decreasing levels over time. In Study 2, Vowels et al. found that those higher (versus lower) in attachment anxiety reported increasing depression and anxiety over time. Attachment avoidance did not predict depression or anxiety in either study. The evidence above suggests that insecure attachment, especially attachment anxiety, may be a predictor of poor mental health during the pandemic.

### Attachment styles and social distancing behaviours

The COVID‐19 pandemic context presents several threats to the attachment system, most notably separation from loved ones due to enforced national lockdowns and social distancing measures as well as persistent mortality salience and exposure to illness‐related cues. This context also required individuals to enact prescriptive COVID‐19‐related protective behaviours to prevent infection and/or spreading the disease to others such as hand washing, maintaining a physical distance from others, and wearing face masks.

Attachment style modulates how we respond to stress and threat (Brennan et al., 1998) including separation from loved ones (Ainsworth et al., 1978; Fraley & Shaver, 1998) and cues of danger, such as illness. Consequently, attachment styles are predictive of how people appraise (Meredith et al., 2005) and cope (Krasuska et al., 2018) with symptoms, manage chronic conditions (Ciechanowski et al., 2004) and take preventive measures, including enacting protective health behaviours (Pietromonaco et al., 2013). Moreover, attachment styles have been found to be predictive of the capacity for prosocial behaviour and empathy (Boag & Carnelley, 2016; Mikulincer et al., 2005). Thus, we believe that the requirement to physically and socially distance from others presents the greatest threat to the attachment system that would initiate individual coping responses to regulate this threat, driven by attachment style.

Therefore, attachment style is likely to be a key predictor of the enactment of social distancing in the context of COVID‐19; this is supported by some initial evidence. In a US context, earlier in the pandemic, Lozano and Fraley (2021) examined attachment styles as a predictor of engagement in, and reminding others about, the following protective behaviours: hand washing, social distancing, wearing face masks, refraining from touching face/mouth, and disinfection of items. Attachment avoidance was negatively associated with both engagement in, and reminding others about the behaviours, and attachment anxiety was positively associated with reminding others. Brulin et al. (2022) examined the associations between attachment and adherence to the COVID‐19 regulations in Sweden. Both attachment anxiety and avoidance were associated with nonadherence to authorities' guidelines, such as social distancing and hand washing. While these findings are consistent with attachment theory and are a first attempt to apply and explore attachment to the COVID‐19 context, much work remains to be done to delineate the ways in which individual differences in attachment style affect people's coping and adherence to social distancing behaviours in the pandemic.

### Toward causality in the present research

Prior research evidence is derived from non‐experimental studies, and their authors have understandably refrained from making causal claims about the association between attachment styles, mental health, and adherence to health guidance. The well‐known phrase “correlation is not causation” cautions researchers in the social and health sciences (Hernán, 2018) to be mindful about the scope and confidence of their conclusions when interpreting results obtained from non‐experimental and cross‐sectional studies. Well‐intentioned caution in this regard often means that cross‐sectional data are assumed to tell us nothing about causality. However, recently, several researchers have argued that reluctance to make causal inferences does little to make the interpretations more reliable (Grosz et al., 2020; Hernán, 2018; Rohrer, 2018; Vowels, 2021). It instead results in a conflation of causal and correlational language, a lack of transparency concerning the (causal) assumptions underlying the research, and a reluctance to adopt robust statistical techniques for improving the validity of our analyses (Grosz et al., 2020; Rohrer, 2018; Vowels, 2021). Indeed, such techniques do exist, and a vast array of statistical developments can be applied to the estimation of causal quantities from observational data (Imbens & Rubin, 2015; Pearl, 2009). Furthermore, there exist techniques for estimation of causal quantities given an assumed structure (a process known as causal inference; Pearl, 2009; Tian & Pearl, 2002; van der Laan & Rose, 2011) as well as techniques for estimating the structure itself (a process known as causal discovery; Glymour et al., 2019; Vowels, Camgoz, & Bowden, 2022).

Researchers in the field of causal discovery warn against interpreting the output of such algorithms too literally, and thus they should be used to inform theory rather than overrule it (Vowels, 2021; Vowels, Camgoz, & Bowden, 2022). These methods nonetheless provide a means to validate certain aspects of theories, to explore data for possible causal structures, and thus to help us specify models that reflect the discovered structure, and which can then be used to test hypotheses. In this work, we take advantage of recent progress in the domain of causal discovery, by using a state‐of‐the‐art causal discovery algorithm known as SAM (Kalainathan et al., 2020). Our aim was to develop a causal theoretical model between individual differences in attachment styles (i.e., secure, anxious, and avoidant), social distancing behaviours (i.e., adherence to government guidelines) and mental health outcomes (i.e., loneliness, depression, and anxiety). We then aimed to quantify the causal estimates using a targeted learning approach (van der Laan & Rose, 2011) which sits at the intersection of machine learning and causality. Targeted learning allowed us to estimate the relationships between two target variables (i.e., the causal relationship between attachment anxiety and depression) without making assumptions about the functional form of that relationship (e.g., linear/non‐linear).

### The current research

We hypothesized that those with an insecure attachment style would report greater loneliness, anxiety, and depression compared to those with a secure attachment style; especially so for those with an anxious or fearful attachment style. Furthermore, we expected secure individuals to better adhere to social isolation/physical distancing than those with an insecure attachment style. Taking data from two different time points, we also examine the effects of attachment styles on depression and anxiety over time. We examine these hypotheses in a secondary analysis of data from two waves of the COVID‐19 Psychological Research Consortium Study (C19PRCS), a longitudinal survey tracking changes in behaviour and mental health over the pandemic in a large nationally representative UK sample of adults.

## METHOD

### Participants and procedure

We conducted a secondary analysis of UK data collected in waves 2 and 3 of the longitudinal, internet‐based survey COVID‐19 Psychological Research Consortium Study (C19PRCS). A detailed methodological account of the C19PRCS is available elsewhere (McBride et al., 2021), and the data are publicly available on the OSF [https://osf.io/v2zur/]. Briefly, UK fieldwork of the C19PRC Study was conducted between April/May 2020 for Wave 2 and July/August 2020 for Wave 3. During Wave 2, strict social distancing measures were in place, whereas during Wave 3, many of the measures had been lifted for the summer and restaurants and pubs were open and two households were allowed to meet indoors. Quota sampling was used to recruit a panel of adults who were nationally representative of the UK population in terms of age, sex, and household income. Participants aged 18 years or older at the time of the survey must have been able to complete the survey in English, and residents in the UK. Consenting adults completed the survey online and were reimbursed by Qualtrics for their time. Ethical approval for this research was provided by a UK University Psychology department (Reference number: 033759). During Wave 2, 1406 participants participated in the survey, but some people did not report on their attachment styles and were thus removed from the analyses. The final sample consisted of 1325 individuals in the cross‐sectional analyses and 950 in the longitudinal analyses. The full demographic variables can be found in Table 1. This study was not preregistered.

**TABLE 1:** Demographic characteristics of participants in cross‐sectional and longitudinal analyses.

Demographic variables	Cross‐sectional (*n* = 1325)	Longitudinal (*n* = 950)
Age	*M* = 49.03	*M* = 51.84
	SD = 14.94	SD = 14.45
	Range = 18–88	Range = 18–88
Change in Income from Pre‐pandemic Levels	*M* = −9.5	*M* = −8.8
	SD = 26.3	SD = 24.2
	Range = −100 to 100	Range = −100 to 100

	*n* (%)	*n* (%)
Gender		
Man	683 (51.5%)	521 (54.8%)
Woman	639 (48.2%)	426 (44.8%)
Transgender	1 (.1%)	1 (.1%)
Other	2 (.2%)	2 (.2%)
Relationship status		
Married	641 (48.4%)	478 (50.3%)
Single	305 (23.0%)	208 (21.9%)
Cohabiting	159 (12.0%)	101 (10.6%)
Separated	21 (1.6%)	18 (1.9%)
Divorced	108 (8.2%)	83 (8.7%)
Widowed	34 (2.6%)	27 (2.8%)
In a registered same‐sex civil partnership	6 (.5%)	4 (.4%)
In a relationship but not living together	51 (3.85)	31 (3.3%)
Number of children		
No	1002 (75.6%)	766 (80.6%)
Yes	323 (24.4%)	184 (19.4%)
Education		
No qualifications	43 (3.2%)	32 (3.4%)
O‐Level/GCSE or similar	251 (18.9%)	160 (16.8%)
A‐Level or similar	229 (17.3%)	148 (15.6%)
Technical qualification	129 (9.7%)	97 (10.2%)
Undergraduate degree	378 (28.5%)	304 (32.0%)
Diploma	72 (5.4%)	46 (4.8%)
Postgraduate degree	208 15.7%)	150 (15.8%)
Other qualification	15 (1.1%)	13 (1.4%)
Race/Ethnicity		
White British/Irish	1168 (88.2%)	861 (90.6%)
White non‐British/Irish	64 (4.8%)	34 (3.6%)
South Asian	43 (3.2%)	24 (2.5%)
Chinese	15 (1.1%)	9 (.9%)
Caribbean or African	13 (1.0%	9 (.9%)
Arab	3 (.2%)	1 (.1%)
Other	19 (1.4%)	11 (1.2%)
Religion		
Christian	697 (52.6%)	513 (54.0%)
Muslim	34 (2.6%)	16 (1.7%)
Jewish	10 (.8%)	8 (.8%)
Hindu	7 (.5%)	4 (.4%)
Buddhist	11 (.8%)	7 (.7%)
Sikh	7 (.5%)	5 (.5%)
Atheist	318 (24.0%)	228 (24.0%)
Agnostic	163 (12.3%)	122 (12.8%)
Other religious conviction	78 (5.9%)	47 (4.9%)
Employment		
Full time	720 (54.3%)	415 (43.7%)
Part time (regular hours)	152 (11.5%)	107 (11.3%)
Zero hours contract	23 (1.7%)	14 (1.5%)
Other flexible work practice	29 (2.2%)	22 (2.3%)
Unemployed (because of coronavirus)	36 (2.7%)	26 (2.7%)
Unemployed (not because of coronavirus)	204 (15.4%)	128 (13.5%)
Retired	272 (20.5%)	238 (25.1%)
Keyworker		
No	940 (70.9%)	703 (74.0%)
Yes	385 (29.1%)	247 (26.0%)
Chronic illness		
No	1004 (75.8%)	710 (74.7%)
Yes	321 (24.2%)	240 (25.3%)
Pregnant (self or partner)		
No	1301 (98.2%)	940 (98.9%)
Yes	24 (1.8%)	10 (1.1%)

### Measures

Due to the space limitations, a detailed description of all variables can be found in Supplemental Material. Attachment style was measured using the Relationships Questionnaire (Bartholomew & Horowitz, 1991), which is a categorical measure of the four attachment styles: secure, anxious, avoidant, and fearful avoidant. Social distancing practices, in accordance with government guidelines during the first UK lockdown, were assessed using a list of 16 statements with respect to the past week. The Generalized Anxiety Disorder Scale (GAD‐7; Spitzer et al., 2006) was used to measure generalized anxiety and the Patient Health Questionnaire (PHQ‐9; Kroenke et al., 2002) depression. Loneliness was measured using a 3‐item Loneliness Scale (Hughes et al., 2004). We also included a set of variables discussed among the study authors and determined to be theoretically causally related to the central variables in the study that we controlled for in the models. These variables include demographics, COVID‐19‐related anxiety and perceived 1 month risk, and hygiene practices.

### Data analysis

We used a state‐of‐the‐art causal discovery algorithm known as Structural Agnostic Modeling (SAM; Kalainathan et al., 2020) to infer the cross‐sectional structure for Wave 2 (17 variables and 1325 participants) and the longitudinal structure across Wave 2 and Wave 3 (19 variables from 895 participants). We included all variables that were expected to be causally linked to the main variables of interest and thus affect the estimation of the causal relationships. We applied a constraint preventing the discovery of causal effects backwards in time, as well as constraints preventing causal links between certain demographics: age and gender cannot be effects; change in income was measured as the change between Waves 1 and 2 and thus was prevented from affecting all demographic variables. We then used a state‐of‐the‐art method at the intersection of machine learning and causality known as targeted learning (van der Laan & Rose, 2011) to estimate the specific effect of attachment styles on social distancing behaviours and mental health outcomes. Details of the data analysis can be found in Supplemental Material.

## RESULTS

Table 2 presents the means and standard deviations as well as the bivariate correlations between the main study variables. The number of people identifying as secure (*n* = 441), avoidant (*n* = 392), and anxious (*n* = 367) was comparable, with fewer people identifying as fearful avoidant (*n* = 124).

**TABLE 2:** Means, standard deviations, and correlations with confidence intervals for wave 2.

Variable	*M*	*SD*	1	2	3	4	5	6	7
1. Secure (*n* = 441)	.33	.47							
2. Fearful (*n* = 124)	.28	.45	−.44** [−.48, −.39]						
3. Anxious (*n* = 367)	.09	.29	−.23** [−.28, −.18]	−.20** [−.25, −.15]					
4. Avoidant (*n* = 392)	.30	.46	−.46** [−.50, −.41]	−.40** [−.45, −.36]	−.21** [−.26, −.16]				
5. Social distancing	12.84	5.94	−.03 [−.09, .02]	.13** [.08, .18]	.09** [.04, .15]	−.15** [−.21, −.10]			
6. Depression	5.25	5.94	−.16** [−.21, −.11]	.21** [.16, .27]	.10** [.05, .15]	−.11** [−.16, −.05]	.31** [.26, .36]		
7. GAD	4.41	5.38	−.15** [−.21, −.10]	.21** [.16, .26]	.11** [.05, .16]	−.12** [−.17, −.06]	.26** [.21, .31]	.85** [.84, .87]	
8. Loneliness	4.73	1.81	−.21** [−.26, −.15]	.26** [.21, .31]	.11** [.05, .16]	−.11** [−.16, −.05]	.17** [.11, .22]	.59** [.56, .63]	.53** [.49, .57]

*Note*: *M* and *SD* are used to represent mean and standard deviation, respectively. Values in square brackets indicate the 95% confidence interval for each correlation. GAD, generalized anxiety disorder. * indicates *p* < .05. ** indicates *p* < .01. Secure: *n* = 441.

### Cross‐sectional model (wave 2)

**FIGURE 1:**
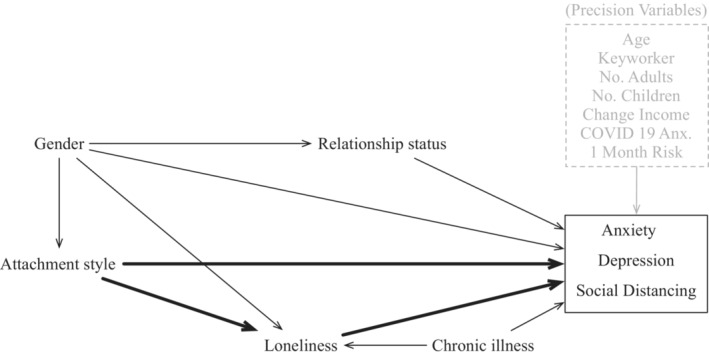
Cross‐sectional causal discovery results. The directed causal relationships between the cause and effect with a probability of at least .5 have been included in the graphs. We have highlighted the theoretically relevant relationships in bold but have included the required control variables in the graph. Precision variables are included in grey as they are not necessary to produce an unbiased estimate but can make the standard errors tighter because they explain variance in the outcome variables.

**FIGURE 2:**
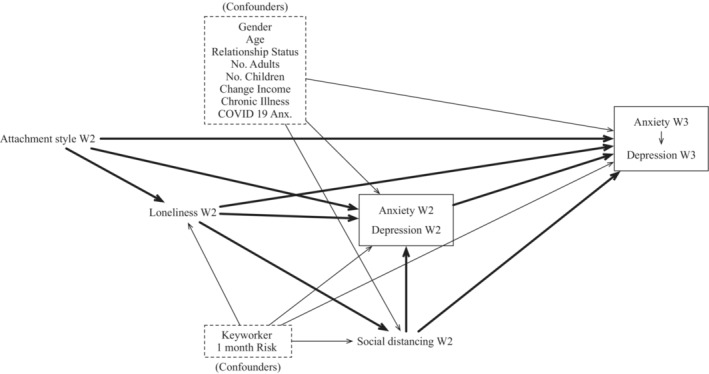
Longitudinal causal discovery results. The directed causal relationships between the cause and effect with a probability of at least .5 have been included in the graphs. We have highlighted the theoretically relevant relationships in bold but have included the required control variables in the graph. There were no precision variables in the longitudinal model given that all the cross‐sectional precision variables caused Wave 2 outcomes as well as Wave 3 outcomes, meaning they introduced confounding in the data and needed to be included as controls.

The graphical illustration of the results from the causal discovery algorithm can be found in Figure 1 for the cross‐sectional data and Figure 2 for the longitudinal data. The full adjacency matrices with all causal paths can be found in the Figures S2 and S3. The directed causal relationships between the cause and effect with a confidence score of at least .5 (where this score ranges between 0 and 1) have been included in the graphs. We can see from Figure 1 that the only putative cause for attachment styles is participants' gender whereas attachment styles cause relationship status, anxiety, depression, loneliness, and social distancing behaviours. Loneliness was identified as a mediator between attachment styles and anxiety, depression, and social distancing behaviours. We have highlighted the theoretically relevant relationships in bold but have also included the required control variables in Figure 1. Precision variables are included in grey as they are not necessary to produce an unbiased estimate but can make the standard errors tighter because they explain variance in the outcome variables.

The algorithm does not provide the direction or the size of the effects and thus we used targeted learning to estimate the causal effects between attachment styles and each of the outcome variables. For the targeted learning, we used only the control variables that were essential in providing accurate causal estimates in line with what are known as the d‐separation rules for causal graphs, and precision variables that can help provide tighter estimates of the effect (i.e., smaller standard errors; Cinelli et al., 2022). Thus, our set of control variables consisted of gender as a confounding variable and age, chronic illness, number of children in the household, adults in the household, change in income, keyworker status, 1 month risk, COVID‐19 anxiety, and pregnancy as precision variables for all outcomes, except for loneliness. For loneliness, we used gender as a control variable but only change in income, keyworker status, 1 month risk, COVID‐19 anxiety, and chronic illness as precision variables.

Table 3 shows both the naive estimates as well as the estimates following the targeted learning steps. The naive estimates are essentially the correlations between the two variables without any control variables. We only describe the targeted learning results below. We use ψ* to denote the targeted learning estimates within the text. The estimates are scaled to be between −1 and +1 so an estimate can be understood in terms of percentages. For example, a ψ* = .10 means a 10% difference in the outcome between two groups. Note that we have opted to maintain a correlational language given that it makes the description of the results easier and readers are more used to interpreting this type of language. Thus, while the language used below is not explicitly causal, we are nonetheless intending for these quantities to be interpreted causally as a change in attachment style *causing* a corresponding change in the outcome.

**TABLE 3:** The cross‐sectional and longitudinal results from the targeted learning analyses.

Group (0 = secure)	Anxiety			Depression			Loneliness			Social distancing behaviours		
	Effect	SE	*p*	Effect	SE	*p*	Effect	SE	*p*	Effect	SE	*p*
Cross‐sectional												
Naïve estimates												
Fearful	.14	.02	<.001	.13	.02	<.001	.21	.02	<.001	.04	.01	.001
Anxious	.14	.02	<.001	.12	.02	<.001	.19	.02	<.001	.05	.01	.005
Avoidant	.01	.02	.541	.01	.01	.306	.04	.02	.040	−.03	.01	<.001
TL estimates												
Fearful	.06	.01	<.001	.05	.01	<.001	.18	.02	<.001	.01	.01	.386
Anxious	.05	.03	.003	.05	.02	.010	.17	.03	<.001	.00	.02	.970
Avoidant	.01	.01	.240	.01	.01	.248	.05	.02	.009	−.02	.01	<.001
Longitudinal												
Naïve estimates												
Fearful	.15	.02	<.001	.14	.02	<.001						
Anxious	.15	.04	<.001	.13	.03	<.001						
Avoidant	.02	.02	.201	.02	.02	.267						
TL estimates (without W2 control)												
Fearful	.07	.02	<.001	.06	.01	<.001						
Anxious	.06	.02	.007	.05	.02	.022						
Avoidant	.03	.01	.025	.02	.01	.181						
TL estimates (with W2 control)												
Fearful	.02	.01	.206	.01	.01	.320						
Anxious	.02	.02	.463	.00	.02	.797						
Avoidant	.01	.01	.375	.00	.01	.650						

Abbreviation: TL, targeted learning.

We found that compared to secure individuals, fearful‐avoidant individuals were higher in anxiety (ψ* = .06, *p* < .001), depression (ψ* = .05, *p* < .001), and loneliness (ψ* = .18, *p* < .001); but did not report engagement in more social distancing behaviours (ψ* = .01, *p* = .386). The results were similar for attachment‐anxious individuals who were higher in anxiety (ψ* = .05, *p* = .003), depression (ψ* = .05, *p* = .010), and loneliness (ψ* = .17, *p* < .001) than secure individuals; but did not report engagement in more social distancing behaviours (ψ* = .00, *p* = .970). Avoidant individuals only differed from secure individuals in their social distancing behaviours: Avoidant individuals were significantly less likely to engage in social distancing behaviours compared to secure individuals (ψ* = −.02, *p* < .001).

### Longitudinal model

The results from the causal discovery algorithm for the longitudinal model can be found in Figure 2. The results were largely similar to the results of the cross‐sectional model with attachment styles being suggested as putative causes for anxiety, depression, loneliness, and social distancing behaviours. Loneliness was again identified as a mediator between attachment styles and the other outcomes. However, social distancing behaviours were also identified as a mediator between loneliness and anxiety and depression. Attachment styles, anxiety, depression, loneliness, and social distancing behaviours at Wave 2 were also causes of depression and anxiety at Wave 3. Anxiety at Wave 3 was also identified as a cause of depression at Wave 3.

Table 3 shows both the naive estimates as well as the estimates following the targeted learning steps for the longitudinal analyses. The naive estimates do not differ between the analyses with and without controlling for time given that naive estimates are estimated without any control variables (including time). For the targeted learning, we used only the control variables that were needed to provide accurate causal estimates in line with the d‐separation rules for causal inference. Based on these rules, we needed to control for age, gender, relationship status, keyworker status, number of adults in the household, number of children in the household, change in income, chronic illness, COVID‐19 anxiety, and perceived 1 month risk.We did not include any mediators in the models as we were interested in the total effect of attachment styles on the mental health outcomes. We present the results for the longitudinal estimates with and without controlling for Wave 2 reports of the variables. The estimates without the Wave 2 control refer to how much we can still explain the mental health outcomes at Wave 3 by the participants' self‐reported attachment style at Wave 2. The estimates including the Wave 2 control variables refer only to changes in the mental health outcomes from Wave 2 to Wave 3 as a result of attachment styles. However, we would not expect a fixed variable (i.e., a variable which is assumed to not change over time and does not in our models) to cause changes in an outcome over time but have included it in case readers are interested in this outcome.

We found that compared to secure individuals, fearful‐avoidant individuals were higher in anxiety (ψ* = .07, *p* < .001) and depression (ψ* = .06, *p* < .001) at Wave 3. We also found that compared to secure individuals, anxious individuals were higher in anxiety (ψ* = .06, *p* = .007) and depression (ψ* = .05, *p* = .022) at Wave 3. Finally, avoidant individuals differed significantly from secure individuals only in that they reported higher anxiety (ψ* = .03, *p* = .025) but not higher depression (ψ* = .01, *p* = .181). Changes in anxiety or depression scores between Waves 2 and 3 were not significantly different between any of the groups.

## DISCUSSION

The purpose of the present study was to identify putative causal relationships between attachment styles, social distancing behaviours, and mental health outcomes. As hypothesized, attachment insecurity was a risk factor for poor mental health during the COVID‐19 pandemic. Specifically, individuals with fearful avoidant or anxious attachment styles were 5%–6% higher in depression and generalized anxiety and 17%–18% lonelier compared to secure individuals. Avoidant individuals did not differ in their depression or anxiety levels but were also significantly lonelier than secure individuals (albeit by a reduced margin, 5%). The differences in levels of depression and anxiety between attachment anxious and fearful‐avoidant individuals and secure individuals remained constant over time. This pattern is in line with that of recent research, which identifies attachment anxiety, rather than avoidance, as being a risk factor for ongoing mental health issues during the pandemic (Mazza et al., 2021; Moccia et al., 2020; Vowels, Carnelley, & Stanton, 2022). To this we would add that those individuals with a fearful avoidant attachment are similarly at risk.

The causal discovery algorithm also identified loneliness as a partial mediator of the causal path between attachment styles to mental health outcomes. We found that while depression and anxiety are higher in anxious and fearful‐avoidant individuals, they are almost four times higher in loneliness than anxiety and depression compared to secure individuals. Again, this pattern has been observed by other researchers; Vismara et al. (2022) showed that loneliness had a partially mediating role between attachment anxiety and mental health outcomes during the COVID‐19 pandemic in a sample of Italian participants. These results suggest that loneliness is particularly prevalent among anxious and fearful‐avoidant individuals and any interventions that are designed to improve mental health outcomes for individuals with insecure attachment styles should focus on preventing and ameliorating loneliness.

Furthermore, we also examined which attachment styles were causally linked to adherence to social distancing guidelines. In contrast to our hypothesis, we found that avoidant individuals were significantly *less* likely to follow social distancing guidelines compared to secure individuals. Indeed, recent research has also found the same pattern (Brulin et al., 2022; Lozano & Fraley, 2021). In our study, however, there was only a small (2%) difference between avoidant and secure individuals, which is not likely to be meaningful behaviourally. Overall, our results suggest that while insecure individuals have worse mental health outcomes and feel lonelier compared to secure individuals, the causal relationship between attachment styles and social distancing measures, while it exists, is very small.

The present study provided causal evidence of the link between attachment styles and mental health outcomes during the COVID‐19 pandemic. The study was the first to our knowledge to use causal methods to examine these relationships. The results from the causal analyses corroborate previous correlational findings but provide a more accurate estimate of the effect size due to the use of targeted learning, which has been shown to produce estimates that are less biased compared to other methods (Luque‐Fernandez et al., 2018; van der Laan & Rose, 2011). Furthermore, the data were drawn from a nationally representative UK survey, and the results were estimated both cross‐sectionally and over time. Thus, we expect that the effect size estimates are a relatively accurate estimate of the real average estimate in the population.

However, there are also several limitations that should be considered. While we were able to establish causal relationships between our variables of interest using state‐of‐the‐art causal discovery and causal inference algorithms, we did not have access to pre‐pandemic data. Thus, we were only able to establish the causal relationships between attachment styles, social distancing behaviours, and mental health outcomes during the COVID‐19 pandemic, but we do not know whether insecure individuals were particularly at risk due to the pandemic or whether they already had higher levels of mental health problems before the pandemic occurred. One study to our knowledge has examined changes in mental health outcomes as a result of the pandemic with pre‐pandemic and early pandemic data (Vowels, Carnelley, & Stanton, 2022) and showed that individuals higher in attachment anxiety were particularly at risk of worse mental health outcomes over time. However, attachment anxiety has also been linked to worse mental health in general (Mikulincer & Shaver, 2016a, 2016b), and attachment security priming has been linked to lower depressed and anxious mood, suggesting a causal role for attachment (Carnelley et al., 2016, 2018). What is clear is that attachment insecurity is causally linked to poorer mental health outcomes, especially to loneliness.

Another limitation of the study relates to the measure of attachment. As is the case with most large datasets in nationally representative samples, the choice of variables is limited to what is available in the dataset. In our case, the only measure of attachment styles was categorical and measured attachment on two dimensions: attachment anxiety and attachment avoidance. This meant that participants were forced to place themselves into one of the four categories, but there may be a great deal of heterogeneity within the categories. Arguably, a categorical measure of attachment is unlikely to suffer from shared method variance with the outcome variable (i.e., be closely correlated because of some third variable such as mood on the day) because a change of category is likely to require an effect which is larger than that of shared method variance. This argument is further supported by the fact that the estimates were the same strength within and between timepoints. However, it is not clear which measures provide a more accurate depiction of attachment styles overall (Fraley, 2019), and the results may not be directly comparable to those of other studies which use a continuous measure of attachment. Our results should be replicated with attachment dimensional measures, and future research should also examine observed behaviours in addition to self‐report.

Finally, the validity of the estimates proposed to correspond with causal quantities relies on four key assumptions generally described in the causal inference literature (Imbens & Rubin, 2015; Pearl, 2009). The first assumption is that our theory and graph are correct. Of course, this is a strong assumption, and one which is ideally validated under experimental conditions. The second assumption is known as ignorability (also known as conditional exchangeability), which is closely related to the first, and is the assumption that there exist no unobserved confounders which otherwise bias the effect size estimation. The third assumption is that of positivity, which is the assumption that there exist a sufficient number of people in each attachment style group to adequately estimate the effects (i.e., the probability of being in each comparison group is positive/non‐zero). The last is known as the Stable Unit Treatment Value Assumption (SUTVA), which is the assumption that the participants are independent of one another, given their control variables (i.e., that the participants do not influence one another). We expect that the latter two assumptions hold in our sample given the relatively large sample size (which helps with positivity) and the participants being independent of one another (which helps the SUTVA). However, it is more difficult to establish whether the first two assumptions hold. We discussed the variables that were included in the study thoroughly among the authors who are experts in attachment and mental health research and validated the theoretical variables using a causal discovery algorithm. We cannot, however, be certain that there are no unobserved confounders that we should have controlled for. Other researchers may disagree with the variables included, and we encourage them to engage in the process of refining our causal theoretical model.

In conclusion, the present study provided causal evidence of the relationship between attachment insecurity and mental health outcomes during the COVID‐19 pandemic. Specifically, we showed that anxious and fearful‐avoidant individuals have higher scores in depression, generalized anxiety, and loneliness, whereas avoidant individuals have higher scores in loneliness compared to secure individuals. The results of the study imply that focusing on improving feelings of loneliness and isolation in insecurely attached individuals can help ameliorate mental health symptoms in this population. Many countries introduced lockdown and social distancing measures during the pandemic, and these measures are in place periodically in different areas of the world. However, given the burden of social isolation among insecure individuals, it is likely that these measures exacerbate feelings of loneliness. Thus, finding ways to support and maintain social connection is likely to be crucial in ameliorating mental health problems in the population.

## AUTHOR CONTRIBUTIONS

**Laura M. Vowels:** Conceptualization; writing – original draft; methodology; formal analysis. **Matthew J. Vowels:** Writing – original draft; formal analysis; methodology. **Katherine B. Carnelley:** Conceptualization; methodology. **Abigail Millings:** Conceptualization; methodology. **Jilly Gibson‐Miller:** Conceptualization; methodology; funding acquisition; investigation; project administration.

## FUNDING INFORMATION

The research was funded by Economic and Social Research Council (Grant number: ES/V004379/1).

## CONFLICT OF INTEREST STATEMENT

There are no conflicts of interest to disclose.

## Supporting information


Data S1

## Data Availability

The data for the project are available on the Open Science Framework (https://osf.io/v2zur/).
