# Supplementary material for: Toward a causal link between attachment styles and mental health during the COVID‐19 pandemic
Source: Br J Clin Psychol. 2023 Jun 9;62(3):605–20. doi: 10.1111/bjc.12428 (PMC10946758; doi:10.1111/bjc.12428)
Supplement: Supplementary file 1 — Data S1 [file BJC-62-605-s001.docx]

**Supplemental Materials for the Manuscript Entitled: “Toward a Causal Link between Attachment Styles and Mental Health during the COVID-19 Pandemic”**

**Details of Measures Included in the Study**

**Attachment style**. Attachment style was measured using the Relationships Questionnaire (Bartholomew & Horowitz, 1991) which included four statements, one for each attachment style. The participants were asked to “Place a checkmark next to the letter corresponding to the style that best describes you or is closest to the way you are”. The statements included comfort with emotional closeness and dependence on others. (Secure = It is easy for me to become emotionally close to others. I am comfortable depending on them and having them depend on me. I don’t worry about being alone or having others not accept me. Fearful avoidant = I am uncomfortable getting close to others. I want emotionally close relationships, but I find it difficult to trust others completely, or to depend on them. I worry that I will be hurt if I allow myself to become too close to others.  Anxious = I want to be completely emotionally intimate with others, but I often find that others are reluctant to get as close as I would like. I am uncomfortable being without close relationships, but I sometimes worry that others don’t value me as much as I value them.  Avoidant = I am comfortable without close emotional relationships. It is very important to me to feel independent and self-sufficient, and I prefer not to depend on others or have others depend on me).

**Social distancing behaviors.** Social distancing practices, in accordance with government guidelines during the first UK lockdown, were assessed using a list of 16 statements with respect to the past week, e.g., “Met up with friends or extended family (outside of your home).” Response scales were: not at all; 1-2 days per week; 3-4 days per week; most days; every day. The social distancing items were coded such that higher scoring reflected greater endorsement of social distancing practices, e.g., the item “Engaged in close contact greetings with people outside of your family (e.g., shaking hands, hugging)” was reversed-scored. We performed an exploratory factor analysis to examine the scale items. The results showed that 10/16 variables loaded well on one factor and were thus included as a total score. Variables that did not cluster well with others included variables about keeping a distance, washing hands straight away, and behaviors that were within guidelines. The reliability of the 10-item scale was α = .91.

**Mental health outcomes.**

**Generalized Anxiety.** Symptoms of GAD were measured using the Generalized Anxiety Disorder 7-item Scale (GAD-7; Spitzer et al., 2006). The GAD-7 has been shown to produce reliable and valid scores in community studies, and the reliability in the current sample was high (α = .94).

**Depression.** Depression was measured using the Patient Health Questionnaire PHQ-9: Kroenke et al., 2002). The PHQ-9 is a 9-item self-report measure that asks participants the degree to which they have been bothered by depressive symptoms in the last two weeks (items are rated on a 3-point Likert scale ranging from 0 [*not bothered at all*] to 2 [*bothered a lot*]). Multiple previous studies attest to the reliability and validity of the PHQ-9 (Hinz et al., 2017). The reliability of the scale in the current sample was α = .93.

**Loneliness.** Loneliness was measured using a 3-item Loneliness Scale (Hughes et al., 2004). Example items include “How often do you feel that you lack companionship?” The items were measured on scale from 1(*Hardly ever*), to 2 (*Some of the time*), to 3 (*Often*). The reliability of the scale in the current sample was α = .87.

**Control variables.**

We also included a set of variables that were theoretically causally related to the central variables in the study that we controlled for in the models. These variables include demographics, COVID-19 related anxiety and perceived one month risk, and hygiene practices and are described below in more detail.

**Demographics.** The following demographic variables were measured at Wave 2 and included in the analyses: age, gender, relationship status, key/essential worker status, number of adults living in household, number of children living in household, change in monthly household income during pandemic (self-reported change with a slider from 100% less to 100% more from compared to before pandemic levels), and currently pregnant – self (partner). Religion, ethnicity, employment status, and education were measured at Wave 1 and only used for descriptive purposes.

**COVID-19-related anxiety and perceived one month risk.** We also included COVID-19 related anxiety and perceived one month risk in the analyses as control variables. The survey included a question “How anxious are you about the coronavirus COVID-19 pandemic?”. The one-month risk included a question “What do you think is your personal percentage risk of being infected with the COVID-19 virus over the following time periods? - In the next month”. Both items were rated on a ‘slider’ (electronic visual analogue scale) to indicate their degree of anxiety/perceived risk with ‘0’ and ‘100’ at the left- and right-hand extremes respectively, and 10-point increments. This produced continuous scores ranging from 0 to 100 with higher scores reflecting higher levels of COVID-19-related anxiety or higher perceived risk.

**Hygienic practices*.*** Reasons for maintaining hygiene practices included 18 self-reported statements (e.g., “I knew about why it was important and had a clear idea about how the virus was transmitted” and “I was able to overcome the physical and/or mental barriers that might have stopped me from doing it”). Response scales were 1 (*strongly disagree*) to 5 (*strongly agree*). We performed an exploratory factor analysis to examine the scale items. The results showed that 11/18 variables loaded well on one factor and were thus included as a total score. Variables that did not cluster well with others included variables that focused on reminders and support and social pressure to engage in hygiene behaviors. The reliability of the 11-item scale was α = .93.

**Full Description of the Data Analysis**

**Data Analysis**

We used a state-of-the-art causal discovery algorithm known as Structural Agnostic Modeling (SAM; Kalainathan et al., 2020). The algorithm is based on a Generative Adversarial Network (Goodfellow et al., 2014) in which one neural network (the adversary) proposes estimations of conditional distributions, and another one (the discriminator) tries to distinguish the estimates from the true data. During optimization, the adversary learns to approximate the true distributions such that the discriminator fails to distinguish the estimates from the originals. The overall model derives a structure that maximizes the fit to the data, whilst enforcing a constraint which encourages acyclicity (no feedback loops in the resulting graph), and a constraint which encourages sparsity. SAM also takes advantages of a number of structural heuristics which can be used to orient cause-effect directions, thus improving over other contemporary approaches for which model fit statistics are known be insufficient for estimating causal directionality (Pearl, 2009; Vowels et al., 2022). By leveraging these heuristics, SAM is thus able to estimate the causal structure of a set of variables under a number of assumptions. The algorithm does not, for example, infer hidden variables for us. In the presence of unobserved confounding, the algorithm may therefore mistake the direction of a causal effect. Nonetheless, it can be used to ‘fill in the gaps’ of our theories, by proposing structures about which we may have no prior domain expertise. These techniques are not meant to overrule our domain expertise, and so we must evaluate the putative structure for face validity. The validity of the subsequent analysis, which itself is informed by the structure, rests on the assumption that this structure is sufficiently correctly specified. Of course, in reality there may exist some key factors without which our analyses become biased. However, given that we are using causal discovery to specify our structure in addition to domain expertise (whereby the latter is usually applied on its own) we would argue that this approach helps us to robustify the specification of our model and therefore our analysis. In particular, knowledge of the structure helps to guide us when it comes to the selection of good control variables for the estimation of causal effects, and it is well known that the selection of ‘bad’ control variables can have a dramatic impact on the resulting estimates (Cinelli et al., 2022; Vowels, 2021).

With regards to the specifics of the analysis, we used SAM to infer the cross-sectional structure for Wave 2 (17 variables and 1325 participants), as well as the longitudinal structure across Wave 2 and Wave 3 (19 variables from 895 participants). We included all variables that were expected to be causally linked to the main variables of interest and thus affect the estimation of the causal relationships. We applied a constraint preventing the discovery of causal effects backwards in time, as well as constraints preventing causal links between certain demographics: age and gender cannot be effects; change in income was measured as the change between Waves 1 and 2 and thus was prevented from affecting all demographic variables.

SAM is a continuously optimized method and can be randomly initialized with a set of starting parameters. This means it does not necessarily converge to the same solution for each initialization. We therefore fit SAM 50 times and took the consensus across the 50 resulting structures. We explored a number of learning rates (0.01 and 0.001), and applied a regularizing penalty of 0.05 which encourages the structure to be acyclic. We found that a learning rate of 0.001 did not converge (the resulting structure was fully saturated), and therefore used the results derived using a learning rate of 0.01. Otherwise, the default hyperparameter settings for SAM were used (see Supplementary Material for a full list of parameters).

The output of SAM is a Directed Acyclic Graph, which is a structured / graphical model encoding the directions of causal influence without cycles. This structure was used to construct a Structural Equation Model (SEM) using the *lavaan* package in R. We used only observed variables rather than constructing latent variables of our constructs given the causal discovery algorithm was conducted with observed variables only. The numbers of paths or ‘edges’ between variables in the cross-sectional and longitudinal graphs were prohibitively high, and the SEM with all variables identified as causally linked to a part of the model would not converge. We thus employed `d-separation’ rules (Koller & Friedman, 2009; Spirtes et al., 2000) to reduce the complexity of the graph without impacting the essential structure. An example of the application of the rules can be simply demonstrated by considering the structure A -> B -> C. Imagine we are concerned with estimating the effect of B on C, then there is no need to estimate the effect of A on B, and the path A -> B can therefore be removed from the model. This is because of what is known as the Markovicity assumption, which tells us that knowing A tells us nothing about C which is not already contained in B. Formally, the statement is that A is independent of C given B. Similarly, if we are only interested in the effect of A on C, we can ignore B - a process known as projection (Glymour, 2001; Richardson et al., 2012). These rules can be applied to all the paths in the full graph and used to identify non-causal paths which otherwise affect the estimation of the paths we care about. They can also be used to identify what are known as ‘precision variables’ which may help in improving the precision of estimation (i.e., to reduce the standard error). Unlike confounders, which are essential to control for, precision variables do not help us debias the estimate of the causal effect. Using these d-separation rules, we can therefore identify variables that are important and variables that can be ignored, and as a result the graph can then be simplified to leave only what is necessary to answer our research questions. Figure S1 illustrates an example structure comprising a cause *T***,** an outcome of interest *Y*, and sets of confounders ***C***, mediators ***M***, and precision variables ***P***, as a Directed Acyclic Graph (DAG). Here, we use bold to indicate sets of multiple variables. We can use the putative structure from the causal discovery stage to identify these variables, and therefore specify our model. SEM is a linear estimator and the causal discovery process used was non-linear and given the large number of variables in the models the fit of the SEM was poor. Thus, the SEM results including all mediations are presented in Tables S1 and S2 in the supplemental file.

**Figure S1**

*A Directed Acyclic Graph depicting the various components for consideration.*


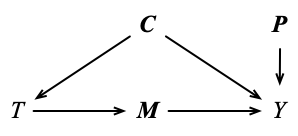


*Note.* We are interested in the effect of T on Y, where Y is the outcome variable; T is the treatment variable; **C** is a set of confounders (which must be controlled for in the model); **P** is a set of precision variables (which do not have to be included but which help explain variance in Y and which can therefore improve estimation precision); **M** is a set of mediations (which should not be included and which can be ignored unless they are of central importance to the research question).

In addition to SEM, we also used a state-of-the-art method at the intersection of machine learning and causality known as targeted learning (van der Laan & Rose, 2011) which has seen myriad applications and demonstrates across a range of subdomains in epidemiology and biostatistics (Li et al. 2022; Luque-Fernandez et al., 2018, Schnitzer et al., 2014). Interested readers are encouraged to consult the accessible introduction by Luque-Fernandez et al. (2018), but essentially the targeted learning frameworks provide us with a means to estimate causal effects of interest without having to make unreasonable assumptions about the functional or parametric form. The effect itself must be unambiguously specified according to the required confounders and precision variables identified from the graph. In the Supplementary Material we provide a list of all included confounders and precision variables used as part of the targeted learning analysis. Targeted learning involves the use of an ensemble of flexible and diverse machine learning algorithms or ‘learners’ to derive an initial estimate for a target causal effect. The ensemble is known as a SuperLearner (van der Laan et al., 2007). It derives estimates from each of the individual learners and, via a process known as k-fold cross-validation, estimates a set of weights across these learners which are used to derive a final linear, weighted combination from each learner. The SuperLearner has been shown to exhibit several desirable properties relating to its optimality and performance. It is more accurate than using any one algorithm alone as it takes a weighted average of many different machine learning algorithms. For modeling the causal effect, we used the following learners for continuous outcome variables: Elastic Net (Zou & Hastie, 2005), Support Vector Regressor (Platt, 1999), linear regressor, linear regressor with quadratic features and moderation effects, Random Forest regressor (Breiman, 2001), a Multilayer Perceptron regressor (Goodfellow et al., 2016), and an AdaBoost Regressor (Drucker, 1997).

The process of targeted learning involves an update step that removes a residual bias associated with the causal estimate derived using the Super Learner, and also renders a Gaussian distribution of estimates which is therefore amenable to the derivation of confidence intervals and *p*-values. The update step requires a second Super Learner model for the cause itself, known as a propensity score model. The propensity score is the likelihood of receiving treatment, and this quantity can be used to help us remove confounding associated with treatment group imbalance. In our case, we were interested in the effect of attachment style (a categorical variable) on a number of continuous outcomes, and thus one can consider attachment style to be equivalent to the treatment in our cause-effect model. The propensity score Super Learner comprises the following algorithms for categorical outcomes: a logistic regressor, a logistic regressor with quadratic and moderation effects, a MultiLayer Perceptron, a Random Forest classifier, a Support Vector classifier, and an AdaBoost classifier. The propensity model is used to generate predictions for the probability of being in a particular attachment category, from a set of predictors. Using the propensity scores, we can derive what is known as a ‘clever covariate’ which quantifies the degree to which the estimate of the causal effect of interest is being biased by the relationship between a set of covariates and the cause. Definitions of clever covariates falls beyond the scope of this paper, but again, interested readers are encouraged to consult Luque-Fernandez et al. (2018). Once we have modeled this bias, we can correct for it by updating the initial estimate. It also provides us with a means to derive the Influence Function (Hampel, 1974), which in turn is used to undertake valid statistical inference, despite the fact that our original estimates were derived using non-parametric methods. The power of the targeted learning approach is thus threefold: We can use powerful non-parametric machine learning algorithms to achieve high precision estimates; we can undertake typical statistical inference; and the update step removes residual bias thus improving the estimate. All algorithms in the Super Learner were implemented using the default implementations in the sklearn package (Pedrogosa et al., 2011).

We concern ourselves with the estimation of the Average Causal Effect (ACE), which is the average difference in outcomes for participants of different treatment groups. For instance, the ACE for people in group 1 compared with group 0 can be expressed as

$\psi_{1,0}=E\left[ E\left[ Y | C=c,P=p,T=1 \right]-E\left[ Y | C=c,P=p,T=0 \right] \right]$,

where E indicates an expectation operator and the bold font denotes that these may be sets of multiple variables. As is usual in causal inference, the validity of our estimates for $\psi$ rests on three key assumptions (Imbens et al., 2015; Pearl, 2009): (1) ignorability - we assume that we have sufficiently controlled for confounding such that we can assume that there exist no remaining unobserved confounders, (2) positivity - we assume that the probability of having any attachment style is bounded away from 0 for all participants, and (3) stable unit treatment value assumption - we assume that the outcomes for each participant are independent of the outcomes for any other participant.

**Figure S2**

*Cross-Sectional Results for the Causal Discovery Algorithm
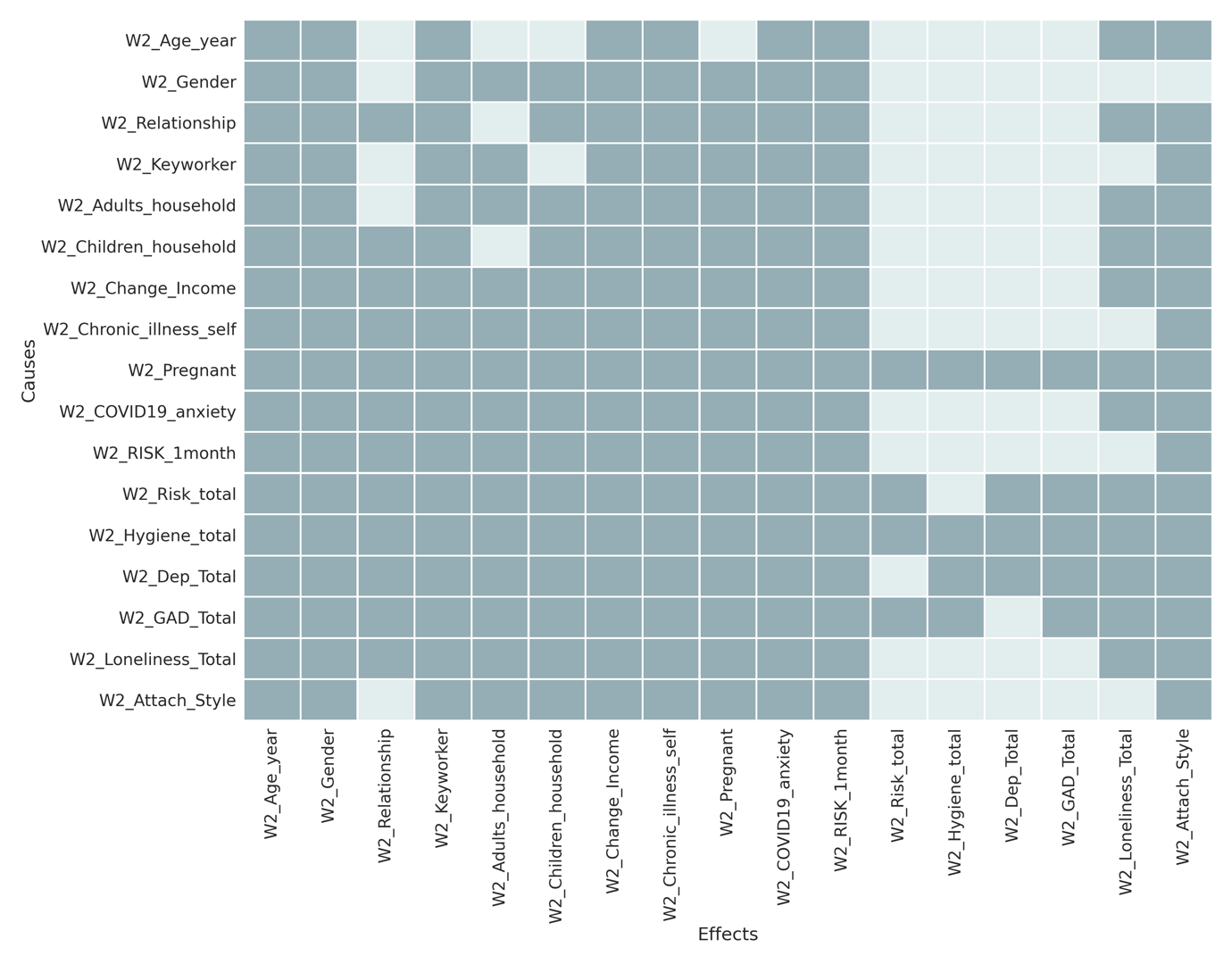
*

*Note.* The causes can be found on the Y axis and effects on the X axis. The boxes in lighter colors identify a directed causal relationship between the cause and effect with a probability of at least 0.5. For example, we see from the figure that the only cause for attachment styles is participants’ gender (there is a lighter colored box with gender on Y axis and attachment style on X axis) whereas attachment styles cause relationship status, anxiety, depression, loneliness, and social distancing behaviors. Attach_Style = attachment style; Dep_Total = depression, GAD_Total = generalized anxiety, Risk_total = social distancing behaviors.

**Figure S3**

*Longitudinal Results for the Causal Discovery Algorithm*

**
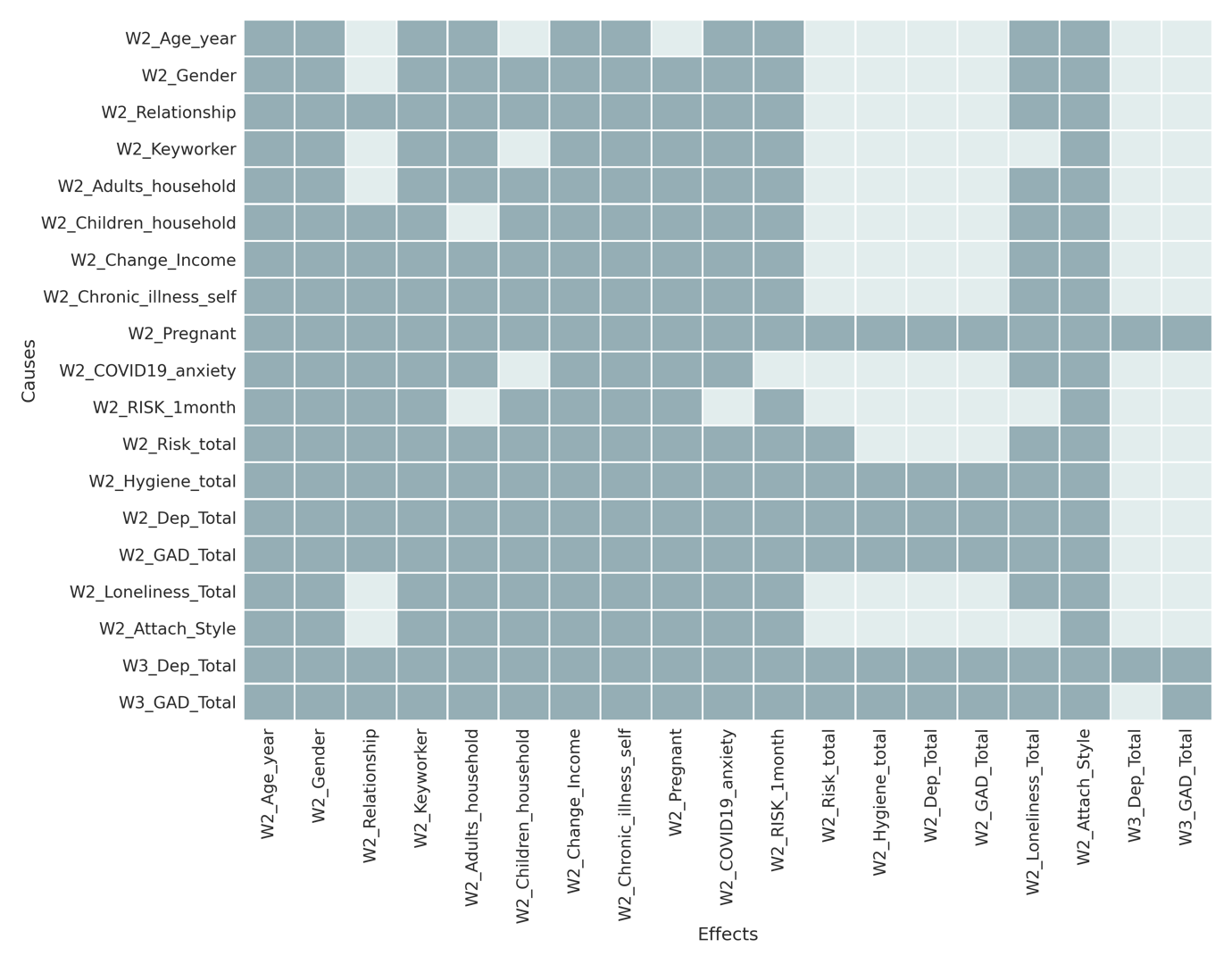
**

*Note.* The causes can be found on the Y axis and effects on the X axis. The boxes in lighter colors identify a directed causal relationship between the cause and effect with a probability of at least 0.5. Attach_Style = attachment style; Dep_Total = depression, GAD_Total = generalized anxiety, Risk_total = social distancing behaviors.

**Results for Structural Equation Modeling**

In addition to the targeted learning approach, we also conducted a more traditional structural equation modeling (SEM) approach which also allowed us to estimate loneliness as a potential mediator. However, SEM is limited in its linear assumptions and given the causal estimates were derived using a non-linear model, the results of the SEM are thus presented in the supplemental file. The full results of the SEM model can be found in Table S1 and the significant results of the relevant variables in Figure S3. The model fit the data well: χ^2^(12) = 35.99, p < .000, CFI = 0.99, TLI = 0.96, RMSEA = 0.04. Based on a sensitivity power analysis, we had a power of .80 to detect a minimum effect size of *r* = .12 and a power of 1.00 to detect a medium effect of *r* = .30 with an alpha level of .05. We found that compared to secure individuals, fearful-avoidant individuals were higher in anxiety (*B* = 1.19, *p* = .001), depression (*B* = 1.10, *p* = .001), and loneliness (*B* = 1.28, *p* < .001); and reported engaging in more social distancing behaviors (*B* = 1.28, *p* < .001). The results were similar for attachment-anxious individuals who were also higher in anxiety (*B* = 1.35, *p* = .003), depression (*B* = 1.18, *p* = .014), and loneliness (*B* = 1.11, *p* < .001); and reported engaging in more social distancing behaviors (*B* = 1.42, *p* = .013) compared to secure individuals. The results for avoidant attachment looked somewhat different with no significant differences between avoidant and secure individuals on anxiety (*B* = 0.07, *p* = .825) or depression (*B* = 0.11, *p* = .741). Avoidant individuals were higher than secure individuals in loneliness (*B* = 0.29, *p* = .014) and reported engaging in less social distancing behaviors (*B* = -1.01, *p* = .009).

There was a significant mediation by loneliness on the causal relationship between attachment styles and the other four outcomes (depression, anxiety, risk, and hygiene behaviors). There was a significant indirect effect through loneliness between fearful-avoidant attachment and anxiety (*B* = 1.74, *p* < .001), depression (*B* = 2.23, *p* < .001), and social distancing behaviors (*B* = 0.40, *p* = .001). There was also a significant indirect effect through loneliness between anxious attachment and anxiety (*B* = 1.54, *p* < .001) and depression (*B* = 1.97, *p* < .001), as well as social distancing behaviors (*B* = 0.36, *p* = .002). There was also a small but significant indirect effect through loneliness between avoidant attachment and anxiety (*B* = 0.40, *p* = .015), depression (*B* = 0.52, *p* < .015), and social distancing behaviors (*B* = 0.09, *p* = .043). All total effects between all causes and effects were significant except the relationship between attachment avoidance and anxiety and depression.

**Figure S4**

*The SEM Results for the Relevant Significant Paths for Cross-Sectional Analyses*

*Note.* Only the main variables of interest were included in the figure. The full results can be found in the tables.

For the longitudinal models, there was a significant effect of fearful-avoidant attachment on anxiety at W3 (*B* = 0.92, *p* = .003) but none of the other longitudinal effects of attachment styles on anxiety or depression were significant when accounting for the W2 levels of the outcome variables. Thus, attachment styles did not cause a significant change from W2 to W3 in anxiety or depression except for fearful-avoidant attachment on anxiety.

**Table S1.**

The Results of the Structural Equation Modeling for Cross-Sectional Data

|  | Model | | | |
| --- | --- | --- | --- | --- |
|  | Estimate | Std. Err. | z | p |
|  | Regression Slopes | | | |
| W2_anxiety |  |  |  |  |
| Fearful | 1.19 | 0.33 | 3.66 | .000 |
| Anxious | 1.35 | 0.46 | 2.96 | .003 |
| Avoidant | 0.07 | 0.31 | 0.22 | .825 |
| Loneliness | 1.39 | 0.07 | 19.48 | .000 |
| Woman | 0.50 | 0.24 | 2.03 | .042 |
| Keyworker | 0.02 | 0.27 | 0.06 | .953 |
| Risk.1month | 3.72 | 0.50 | 7.51 | .000 |
| Chronic.illness.self | -0.02 | 0.29 | -0.08 | .932 |
| W2_depression |  |  |  |  |
| Fearful | 1.10 | 0.34 | 3.21 | .001 |
| Anxious | 1.18 | 0.48 | 2.45 | .014 |
| Avoidant | 0.11 | 0.33 | 0.33 | .741 |
| Loneliness | 1.78 | 0.08 | 23.59 | .000 |
| Woman | 0.24 | 0.26 | 0.94 | .345 |
| Keyworker | 0.01 | 0.29 | 0.02 | .982 |
| Risk.1month | 3.39 | 0.52 | 6.47 | .000 |
| Chronic.illness.self | 0.03 | 0.30 | 0.09 | .932 |
| W2_loneliness |  |  |  |  |
| Fearful | 1.25 | 0.12 | 10.37 | .000 |
| Anxious | 1.11 | 0.17 | 6.42 | .000 |
| Avoidant | 0.29 | 0.12 | 2.45 | .014 |
| Woman | 0.15 | 0.09 | 1.60 | .109 |
| Keyworker | 0.15 | 0.10 | 1.43 | .152 |
| Risk.1month | 0.84 | 0.19 | 4.45 | .000 |
| Chronic.illness.self | 0.39 | 0.11 | 3.55 | .000 |
| W2_social distancing |  |  |  |  |
| Fearful | 0.92 | 0.41 | 2.25 | .024 |
| Anxious | 1.42 | 0.57 | 2.49 | .013 |
| Avoidant | -1.01 | 0.39 | -2.61 | .009 |
| Loneliness | 0.32 | 0.09 | 3.60 | .000 |
| Woman | -0.97 | 0.31 | -3.18 | .001 |
| Keyworker | 2.41 | 0.34 | 7.04 | .000 |
| Risk.1month | 3.91 | 0.62 | 6.29 | .000 |
| Chronic.illness.self | 0.03 | 0.36 | 0.08 | .936 |
| W2_hygiene |  |  |  |  |
| Fearful | -2.84 | 0.52 | -5.48 | .000 |
| Anxious | -4.42 | 0.73 | -6.10 | .000 |
| Avoidant | -0.97 | 0.49 | -1.98 | .048 |
| Loneliness | -0.12 | 0.11 | -1.05 | .293 |
| Woman | 1.74 | 0.39 | 4.46 | .000 |
| Keyworker | -1.37 | 0.44 | -3.15 | .002 |
| Risk.1month | -0.99 | 0.79 | -1.25 | .211 |
| Chronic.illness.self | -0.55 | 0.46 | -1.20 | .229 |
|  | Intercepts | | | |
| Anxiety | -4.40 | 0.42 | -10.55 | .000 |
| Depression | -5.13 | 0.44 | -11.65 | .000 |
| Loneliness | 3.64 | 0.12 | 29.20 | .000 |
| Risk | 9.40 | 0.52 | 18.01 | .000 |
| Hygiene | 48.89 | 0.66 | 73.65 | .000 |
| Fearful | 0.28 | 0.01 | 22.53 | .000 |
| Anxious | 0.09 | 0.01 | 11.75 | .000 |
| Avoidant | 0.30 | 0.01 | 23.59 | .000 |
| Woman | 0.48^+^ |  |  |  |
| Keyworker | 0.29^+^ |  |  |  |
| Risk.1month | 0.40^+^ |  |  |  |
| Chronic.illness.self | 0.24^+^ |  |  |  |
|  | Indirect Effects through Loneliness | | | |
| Fearful to anxiety | 1.74 | 0.19 | 9.15 | .000 |
| Anxious to anxiety | 1.54 | 0.25 | 6.10 | .000 |
| Avoidance to anxiety | 0.40 | 0.17 | 2.43 | .015 |
| Fearful to depression | 2.23 | 0.23 | 9.49 | .000 |
| Anxious to depression | 1.97 | 0.32 | 6.20 | .000 |
| Avoidance to depression | 0.52 | 0.21 | 2.43 | .015 |
| Fearful to social distancing | 0.40 | 0.12 | 3.40 | .001 |
| Anxious to social distancing | 0.36 | 0.11 | 3.14 | .002 |
| Avoidance to social distancing | 0.09 | 0.05 | 2.02 | .043 |
|  | Total Effects | | | |
| Total.fear.anxiety | 2.93 | 0.36 | 8.25 | .000 |
| Total.anx.anxiety | 2.89 | 0.51 | 5.68 | .000 |
| Total.avo.anxiety | 0.47 | 0.35 | 1.35 | .177 |
| Total.fear.depression | 3.33 | 0.39 | 8.44 | .000 |
| Total.anx.depression | 3.15 | 0.56 | 5.58 | .000 |
| Total.avo.depression | 0.63 | 0.39 | 1.61 | .107 |
| Total.fear.social distancing | 1.32 | 0.39 | 3.35 | .001 |
| Total.anx.social distancing | 1.78 | 0.56 | 3.15 | .002 |
| Total.avo.social distancing | -0.92 | 0.39 | -2.36 | .018 |
|  | Fit Indices | | | |
| χ^2^ | 35.99(11) |  |  | .000 |
| CFI | 0.99 |  |  |  |
| TLI | 0.96 |  |  |  |
| RMSEA | 0.04 |  |  |  |
| ^+^Fixed parameter | | | | |

*Note.* Gender, keyworker status, 1 month risk, and chronic illness were used as control variables as identified by the causal discovery algorithm and minSEM.

**Table S2.**

The Results of the Structural Equation Modeling for Longitudinal Data

|  | Model | | | |
| --- | --- | --- | --- | --- |
|  | Estimate | Std. Err. | z | p |
|  | Regression Slopes | | | |
| W3_GAD_Total |  |  |  |  |
| **W2.fearful** | **0.92** | **0.31** | **3.01** | **.003** |
| **W2.anxious** | **0.76** | **0.46** | **1.64** | **.100** |
| **W2.avoidant** | **0.30** | **0.28** | **1.09** | **.276** |
| *W2.Loneliness.Total* | *0.39* | *0.08* | *4.80* | *.000* |
| *W2.Social distancing.total* | *0.00* | *0.02* | *0.18* | *.856* |
| *W2.Hygiene.total* | *-0.01* | *0.02* | *-0.38* | *.706* |
| *W2.GAD.Total* | *0.62* | *0.03* | *21.74* | *.000* |
| W2.Age.year | 0.00 | 0.01 | 0.34 | .735 |
| W2.woman | 0.31 | 0.23 | 1.35 | .177 |
| W2.married | 0.24 | 0.35 | 0.69 | .490 |
| W2.single | 0.61 | 0.39 | 1.57 | .116 |
| W2.cohabiting | 0.61 | 0.46 | 1.32 | .188 |
| W2.Keyworker | -0.10 | 0.27 | -0.37 | .714 |
| W2.Adults.household | 0.09 | 0.14 | 0.66 | .507 |
| W2.Children.household | -0.01 | 0.18 | -0.08 | .936 |
| W2.Change.Income | 0.00 | 0.00 | 0.60 | .552 |
| W2.Chronic.illness.self | 0.46 | 0.26 | 1.80 | .072 |
| W2.COVID19.anxiety | 0.01 | 0.00 | 2.52 | .012 |
| W2.RISK.1month | 0.00 | 0.00 | 0.57 | .566 |
| W3_Dep_Total |  |  |  |  |
| **W2.fearful** | **0.04** | **0.29** | **0.15** | **.880** |
| **W2.anxious** | **-0.24** | **0.43** | **-0.57** | **.571** |
| **W2.avoidant** | **-0.16** | **0.26** | **-0.61** | **.544** |
| *W2.Loneliness.Total* | *0.33* | *0.08* | *4.16* | *.000* |
| *W2.Social_distancing.total* | *0.02* | *0.02* | *1.10* | *.271* |
| *W2.Hygiene.total* | *-0.03* | *0.02* | *-2.08* | *.038* |
| *W2.Dep.Total* | *0.26* | *0.03* | *9.20* | *.000* |
| *W3.GAD.Total* | *0.73* | *0.03* | *25.80* | *.000* |
| W2.Age.year | 0.00 | 0.01 | 0.19 | .849 |
| W2.woman | -0.36 | 0.22 | -1.66 | .098 |
| W2.married | 0.48 | 0.33 | 1.46 | .144 |
| W2.single | 0.04 | 0.36 | 0.11 | .913 |
| W2.cohabiting | -0.21 | 0.43 | -0.49 | .621 |
| W2.Keyworker | -0.43 | 0.25 | -1.71 | .088 |
| W2.Adults.household | -0.24 | 0.13 | -1.76 | .078 |
| W2.Children.household | -0.31 | 0.17 | -1.86 | .063 |
| W2.Change.Income | -0.00 | 0.00 | -0.53 | .595 |
| W2.Chronic.illness.self | -0.02 | 0.24 | -0.10 | .921 |
| W2.COVID19.anxiety | -0.01 | 0.00 | -1.86 | .063 |
| W2.RISK.1month | 0.01 | 0.00 | 1.12 | .263 |
| W2_GAD_Total |  |  |  |  |
| W2.fearful | 0.86 | 0.35 | 2.47 | .013 |
| W2.anxious | 1.15 | 0.52 | 2.20 | .028 |
| W2.avoidant | 0.35 | 0.31 | 1.12 | .264 |
| W2.Loneliness.Total | 1.19 | 0.09 | 13.88 | .000 |
| W2.Social_distancing.total | 0.10 | 0.02 | 4.12 | .000 |
| W2.Hygiene.total | -0.02 | 0.02 | -1.25 | .210 |
| W2.Age.year | -0.05 | 0.01 | -4.49 | .000 |
| W2.woman | 0.55 | 0.26 | 2.08 | .037 |
| W2.married | 0.80 | 0.40 | 2.01 | .044 |
| W2.single | 0.07 | 0.44 | 0.15 | .880 |
| W2.cohabiting | 0.33 | 0.52 | 0.64 | .525 |
| W2.Keyworker | -0.38 | 0.31 | -1.23 | .218 |
| W2.Adults.household | 0.23 | 0.16 | 1.41 | .157 |
| W2.Children.household | -0.54 | 0.20 | -2.63 | .008 |
| W2.Change.Income | -0.01 | 0.01 | -0.95 | .341 |
| W2.Chronic.illness.self | 0.03 | 0.29 | 0.11 | .912 |
| W2.COVID19.anxiety | 0.05 | 0.01 | 9.42 | .000 |
| W2.RISK.1month | 0.01 | 0.01 | 2.49 | .013 |
| W2_Dep_Total |  |  |  |  |
| W2.fearful | 0.88 | 0.37 | 2.36 | .018 |
| W2.anxious | 1.25 | 0.57 | 2.21 | .027 |
| W2.avoidant | 0.45 | 0.34 | 1.33 | .184 |
| W2.Loneliness.Total | 1.49 | 0.09 | 16.10 | .000 |
| W2.Social_distancing.total | 0.15 | 0.03 | 5.68 | .000 |
| W2.Hygiene.total | -0.04 | 0.02 | -1.80 | .071 |
| W2.Age.year | -0.04 | 0.01 | -2.94 | .003 |
| W2.woman | 0.12 | 0.28 | 0.41 | .680 |
| W2.married | 0.49 | 0.43 | 1.13 | .257 |
| W2.single | 0.66 | 0.48 | 1.38 | .169 |
| W2.cohabiting | 0.42 | 0.57 | 0.75 | .455 |
| W2.Keyworker | -0.50 | 0.33 | -1.48 | .139 |
| W2.Adults.household | 0.12 | 0.18 | 0.67 | .503 |
| W2.Children.household | -0.24 | 0.22 | -1.08 | .281 |
| W2.Change.Income | -0.01 | 0.01 | -1.27 | .204 |
| W2.Chronic.illness.self | -0.05 | 0.32 | -0.17 | .864 |
| W2.COVID19.anxiety | 0.03 | 0.01 | 5.55 | .000 |
| W2.RISK.1month | 0.01 | 0.01 | 1.68 | .093 |
| W2_Loneliness_Total |  |  |  |  |
| W2.fearful | 1.31 | 0.14 | 9.37 | .000 |
| W2.anxious | 1.23 | 0.22 | 5.60 | .000 |
| W2.avoidant | 0.30 | 0.13 | 2.28 | .022 |
| W2.woman | 0.16 | 0.11 | 1.44 | .150 |
| W2_Risk_total |  |  |  |  |
| W2.fearful | 0.66 | 0.46 | 1.43 | .152 |
| W2.anxious | 1.77 | 0.69 | 2.55 | .011 |
| W2.avoidant | -0.76 | 0.42 | -1.82 | .069 |
| W2.Loneliness.Total | 0.29 | 0.11 | 2.54 | .011 |
| W2.Age.year | -0.04 | 0.02 | -2.92 | .003 |
| W2.woman | -0.97 | 0.35 | -2.80 | .005 |
| W2.married | 0.48 | 0.53 | 0.91 | .364 |
| W2.single | -0.29 | 0.59 | -0.49 | .622 |
| W2.cohabiting | -0.20 | 0.70 | -0.28 | .779 |
| W2.Keyworker | 1.59 | 0.41 | 3.91 | .000 |
| W2.Adults.household | -0.46 | 0.21 | -2.17 | .030 |
| W2.Children.household | 0.78 | 0.27 | 2.89 | .004 |
| W2.COVID19.anxiety | -0.00 | 0.01 | -0.36 | .716 |
| W2.RISK.1month | 0.03 | 0.01 | 4.57 | .000 |
| W2_Hygiene_total |  |  |  |  |
| W2.fearful | -2.15 | 0.59 | -3.62 | .000 |
| W2.anxious | -2.33 | 0.90 | -2.59 | .010 |
| W2.avoidant | -0.68 | 0.54 | -1.26 | .209 |
| W2.Loneliness.Total | -0.04 | 0.15 | -0.28 | .779 |
| W2.Social_distancing.total | -0.28 | 0.04 | -6.66 | .000 |
| W2.Age.year | 0.06 | 0.02 | 3.20 | .001 |
| W2.woman | 1.99 | 0.45 | 4.44 | .000 |
| W2.married | 0.78 | 0.69 | 1.13 | .257 |
| W2.single | -0.06 | 0.77 | -0.08 | .934 |
| W2.cohabiting | 0.58 | 0.90 | 0.65 | .518 |
| W2.Keyworker | 0.27 | 0.53 | 0.50 | .619 |
| W2.Adults.household | 0.10 | 0.28 | 0.35 | .726 |
| W2.Children.household | -0.37 | 0.35 | -1.05 | .295 |
| W2.Change.Income | -0.01 | 0.01 | -1.22 | .224 |
| W2.Chronic.illness.self | -0.88 | 0.51 | -1.73 | .083 |
| W2.COVID19.anxiety | 0.04 | 0.01 | 4.30 | .000 |
| W2.RISK.1month | -0.01 | 0.01 | -0.74 | .461 |
| W2_Children_household |  |  |  |  |
| W2.Age.year | -0.01 | 0.00 | -8.13 | .000 |
| W2.Keyworker | 0.07 | 0.05 | 1.41 | .158 |
| W2.RISK.1month | 0.00 | 0.00 | 1.58 | .114 |
| W2_Adults_household |  |  |  |  |
| W2.Children.household | 0.03 | 0.04 | 0.85 | .393 |
| W2.RISK.1month | -0.00 | 0.00 | -1.49 | .137 |
| W2_married |  |  |  |  |
| W2.Keyworker | 0.03 | 0.03 | 0.97 | .332 |
| W2.Adults.household | 0.18 | 0.02 | 10.00 | .000 |
| W2.fearful | -0.18 | 0.04 | -4.55 | .000 |
| W2.anxious | -0.10 | 0.06 | -1.64 | .102 |
| W2.avoidant | -0.08 | 0.04 | -2.26 | .024 |
| W2_single |  |  |  |  |
| W2.Keyworker | 0.01 | 0.03 | 0.45 | .652 |
| W2.Adults.household | -0.06 | 0.02 | -4.19 | .000 |
| W2.fearful | 0.16 | 0.03 | 4.73 | .000 |
| W2.anxious | 0.16 | 0.05 | 2.94 | .003 |
| W2.avoidant | 0.11 | 0.03 | 3.44 | .001 |
| W2_cohabiting |  |  |  |  |
| W2.Keyworker | 0.00 | 0.02 | 0.04 | .969 |
| W2.Adults.household | 0.01 | 0.01 | 0.94 | .347 |
| W2.fearful | 0.01 | 0.03 | 0.29 | .768 |
| W2.anxious | -0.01 | 0.04 | -0.25 | .801 |
| W2.avoidant | -0.02 | 0.02 | -0.88 | .380 |
|  | Intercepts | | | |
| W3.GAD.Total | -2.19 | 1.20 | -1.82 | .069 |
| W3.Dep.Total | 1.42 | 1.13 | 1.26 | .206 |
| W2.GAD.Total | -3.95 | 1.36 | -2.89 | .004 |
| W2.Dep.Total | -3.85 | 1.47 | -2.61 | .009 |
| W2.Loneliness.Total | 4.04 | 0.10 | 39.50 | .000 |
| W2.Social_distancing.total | 12.80 | 1.37 | 9.37 | .000 |
| W2.Hygiene.total | 44.89 | 1.85 | 24.26 | .000 |
| W2.Children.household | 0.90 | 0.10 | 9.33 | .000 |
| W2.Adults.household | 2.03 | 0.05 | 38.03 | .000 |
| W2.married | 0.22 | 0.04 | 4.95 | .000 |
| W2.single | 0.26 | 0.04 | 6.67 | .000 |
| W2.cohabiting | 0.09 | 0.03 | 3.06 | .002 |
| W2.fearful | 0.25 | 0.01 | 17.57 | .000 |
| W2.anxious | 0.07 | 0.01 | 8.63 | .000 |
| W2.avoidant | 0.30 | 0.01 | 20.13 | .000 |
| W2.woman | 0.45^+^ |  |  |  |
| W2.Age.year | 51.84^+^ |  |  |  |
| W2.Keyworker | 0.26^+^ |  |  |  |
| W2.Change.Income | -8.80^+^ |  |  |  |
| W2.Chronic.illness.self | 0.25^+^ |  |  |  |
| W2.COVID19.anxiety | 60.41^+^ |  |  |  |
| W2.RISK.1month | 38.50^+^ |  |  |  |
|  | Indirect Effects* | | | |
| *FearfultoLtoGAD* | *0.52* | *0.12* | *4.27* | *.000* |
| *AnxioustoLtoGAD* | *0.49* | *0.13* | *3.65* | *.000* |
| *AvoidancetoLtoGAD* | *0.12* | *0.06* | *2.06* | *.039* |
| *FearfultoLtoDep* | *0.44* | *0.11* | *3.81* | *.000* |
| *AnxioustoLtoDep* | *0.41* | *0.12* | *3.34* | *.001* |
| *AvoidancetoLtoDep* | *0.10* | *0.05* | *2.00* | *.045* |
| *FearfultoLtoHtoGAD* | *0.00* | *0.00* | *0.23* | *.822* |
| *AnxioustoLtoHtoGAD* | *0.00* | *0.00* | *0.22* | *.822* |
| *AvoidancetoLtoHtoGAD* | *0.00* | *0.00* | *0.22* | *.823* |
| *FearfultoLtoHtoDep* | *0.00* | *0.01* | *0.28* | *.781* |
| *AnxioustoLtoHtoDep* | *0.00* | *0.01* | *0.28* | *.781* |
| *AvoidancetoLtoHtoDep* | *0.00* | *0.00* | *0.28* | *.783* |
| *FearfultoLtoRtoHtoGAD* | *0.00* | *0.00* | *0.37* | *.710* |
| *AnxioustoLtoRtoHtoGAD* | *0.00* | *0.00* | *0.37* | *.710* |
| *AvoidancetoLtoRtoHtoGAD* | *0.00* | *0.00* | *0.37* | *.713* |
| *FearfultoLtoRtoHtoDep* | *0.00* | *0.00* | *1.54* | *.123* |
| *AnxioustoLtoRtoHtoDep* | *0.00* | *0.00* | *1.51* | *.132* |
| *AvoidancetoLtoRtoHtoDep* | *0.00* | *0.00* | *1.29* | *.197* |
| *FearfultoRtoGAD* | *0.00* | *0.01* | *0.18* | *.857* |
| *AnxioustoRtoGAD* | *0.01* | *0.04* | *0.18* | *.857* |
| *AvoidancetoRtoGAD* | *-0.00* | *0.02* | *-0.18* | *.857* |
| *FearfultoRtoDep* | *0.02* | *0.02* | *0.87* | *.382* |
| *AnxioustoRtoDep* | *0.04* | *0.04* | *1.01* | *.312* |
| *AvoidancetoRtoDep* | *-0.02* | *0.02* | *-0.94* | *.346* |
| *FearfultoRtoHtoGAD* | *0.00* | *0.00* | *0.36* | *.716* |
| *AnxioustoRtoHtoGAD* | *0.00* | *0.01* | *0.37* | *.710* |
| *AvoidancetoRtoHtoGAD* | *-0.00* | *0.00* | *-0.37* | *.712* |
| *FearfultoRtoHtoDep* | *0.01* | *0.01* | *1.16* | *.245* |
| *AnxioustoRtoHtoDep* | *0.02* | *0.01* | *1.57* | *.117* |
| *AvoidancetoRtoHtoDep* | *-0.01* | *0.01* | *-1.34* | *.180* |
|  | Total Effects | | | |
| total.fear.GAD | 1.44 | 0.31 | 4.64 | .000 |
| total.anx.GAD | 1.25 | 0.47 | 2.68 | .007 |
| total.avo.GAD | 0.42 | 0.28 | 1.48 | .139 |
| total.fear.Dep | 0.51 | 0.29 | 1.72 | .086 |
| total.anx.Dep | 0.20 | 0.44 | 0.46 | .649 |
| total.avo.Dep | -0.05 | 0.26 | -0.17 | .861 |
|  | Fit Indices | | | |
| χ^2^ | 2245.54(77) |  |  | .000 |
| CFI | 0.63 |  |  |  |
| TLI | 0.00 |  |  |  |
| RMSEA | 0.17 |  |  |  |
| ^+^Fixed parameter | | | | |

*Note.* The full model results are presented in the table for transparency but only the bolded paths are of interest. All indirect effects are italicized. The fit effects of this model are poor but given fit statistics are inherently predictive metrics rather than causal, we have not modified the model.

*Indirect effects: L = loneliness, H = hygiene, R = risk behaviors. The indirect paths can be interpreted as: AvoidancetoLtoRtoHtoGAD = indirect affect of attachment avoidance to anxiety through loneliness, risk behavior, and hygiene (serial mediation).

**Model Specification for Targeted Learning**

Outcome: W3_Dep_Total .

Confounders: set() .

Precisions: {'W2_Chronic_illness_self', 'W2_Adults_household', 'W2_Keyworker', 'W2_Change_Income', 'W2_Age_year', 'W2_Children_household', 'W2_RISK_1month', 'W2_COVID19_anxiety', 'W2_Pregnant', 'W2_Gender'}

Outcome: W3_GAD_Total .

Confounders: set() .

Precisions: {'W2_Chronic_illness_self', 'W2_Adults_household', 'W2_Keyworker', 'W2_Change_Income', 'W2_Age_year', 'W2_Children_household', 'W2_RISK_1month', 'W2_COVID19_anxiety', 'W2_Pregnant', 'W2_Gender'}

Outcome: W2_Risk_total .

Confounders: {'W2_Gender'} . Precisions: {'W2_Chronic_illness_self', 'W2_Pregnant', 'W2_Children_household', 'W2_COVID19_anxiety', 'W2_Change_Income', 'W2_Keyworker', 'W2_Age_year', 'W2_RISK_1month'}

Outcome: W2_Dep_Total .

Confounders: {'W2_Gender'} .

Precisions: {'W2_Chronic_illness_self', 'W2_Pregnant', 'W2_Children_household', 'W2_COVID19_anxiety', 'W2_Change_Income', 'W2_Keyworker', 'W2_Age_year', 'W2_RISK_1month'}

Outcome: W2_GAD_Total .

Confounders: {'W2_Gender'} .

Precisions: {'W2_Chronic_illness_self', 'W2_Pregnant', 'W2_Children_household', 'W2_COVID19_anxiety', 'W2_Change_Income', 'W2_Keyworker', 'W2_Age_year', 'W2_RISK_1month'}

Outcome: W2_Loneliness_Total .

Confounders: {'W2_Gender'} .

Precisions: {'W2_Chronic_illness_self', 'W2_COVID19_anxiety', 'W2_Change_Income', 'W2_Keyworker', 'W2_RISK_1month'}

-------------Intentional MEDIATION------------

mediator: 'W2_Dep_Total'

outcome: W3_Dep_Total .

Confounders: set() .

Precisions: {'W2_Chronic_illness_self', 'W2_Adults_household', 'W2_Keyworker', 'W2_Change_Income', 'W2_Age_year', 'W2_Children_household', 'W2_RISK_1month', 'W2_COVID19_anxiety', 'W2_Pregnant', 'W2_Gender'}

mediator: 'W2_GAD_Total'

outcome: W3_GAD_Total .

Confounders: set() .

Precisions: {'W2_Chronic_illness_self', 'W2_Adults_household', 'W2_Keyworker', 'W2_Change_Income', 'W2_Age_year', 'W2_Children_household', 'W2_RISK_1month', 'W2_COVID19_anxiety', 'W2_Pregnant', 'W2_Gender'}
